# Femoral Component Fracture in a Total Knee Arthroplasty Patient With a Persistent Flexion Contracture

**DOI:** 10.1016/j.artd.2023.101174

**Published:** 2023-07-22

**Authors:** Jackson P. Tate, Andrew M. Schneider, Nicholas M. Brown

**Affiliations:** a Loyola University Chicago Stritch School of Medicine, Maywood, IL, USA; b Department of Orthopaedic Surgery and Rehabilitation Medicine, University of Chicago Medical Center, Chicago, IL, USA; c Department of Orthopaedic Surgery and Rehabilitation, Loyola University Medical Center, Maywood, IL, USA

**Keywords:** Flexion contracture, Component fracture, Fatigue failure

## Abstract

This article reports a rare case of a total knee arthroplasty femoral component fracture. Fractures of early knee systems were attributed to design flaws. Modern design failures have been attributed to poor surgical technique or underlying osteolysis. Here, we report a fracture in the Vanguard prosthesis (Zimmer Biomet, Warsaw, IN) 12 years after implantation in a patient with a persistent flexion contracture. The fracture likely occurred due to fatigue failure of the anterior flange secondary to increased stress from a high riding patella. Although femoral component fractures are rare, they should be considered as a potential complication, especially in patients with special load considerations. For these patients, it is essential that the prosthesis be properly supported with clean cuts and an adequate cement mantle.

## Introduction

There have been few reported total knee arthroplasty (TKA) failures due to fracture of the femoral component. Previously reported cases have been attributed to inherent femoral component design flaws, which have been addressed by modern component design [[1], [2], [3], [4], [5]]. Component fracture in modern knee systems is rare, and the etiology of mechanical failure in these components is unclear. However, the available literature hypothesizes that failure occurs due to a fatigue mechanism in the setting of poor surgical technique and underlying osteolysis, which leaves the femoral component with inadequate support and thus vulnerable to fatigue failure [1,[6], [7], [8], [9], [10], [11], [12], [13], [14]].

Here, we report a case of a femoral component fracture at the anterior flange in the Vanguard prosthesis (Zimmer Biomet, Warsaw, IN) 12 years after primary TKA. This fracture was likely due to increased stress from a high riding patella secondary to a persistent flexion contracture. The patient provided informed written consent for data concerning his case to be submitted for publication.

## Case history

Our patient is a 66-year-old male who suffered a traumatic subdural hematoma as a child, which resulted in left-sided partial paralysis and a persistent flexion contracture since the time of injury. He has had multiple surgical and nonsurgical attempts at addressing the 30-degree flexion contracture, but it has always recurred and persisted.

**Figure 1:**
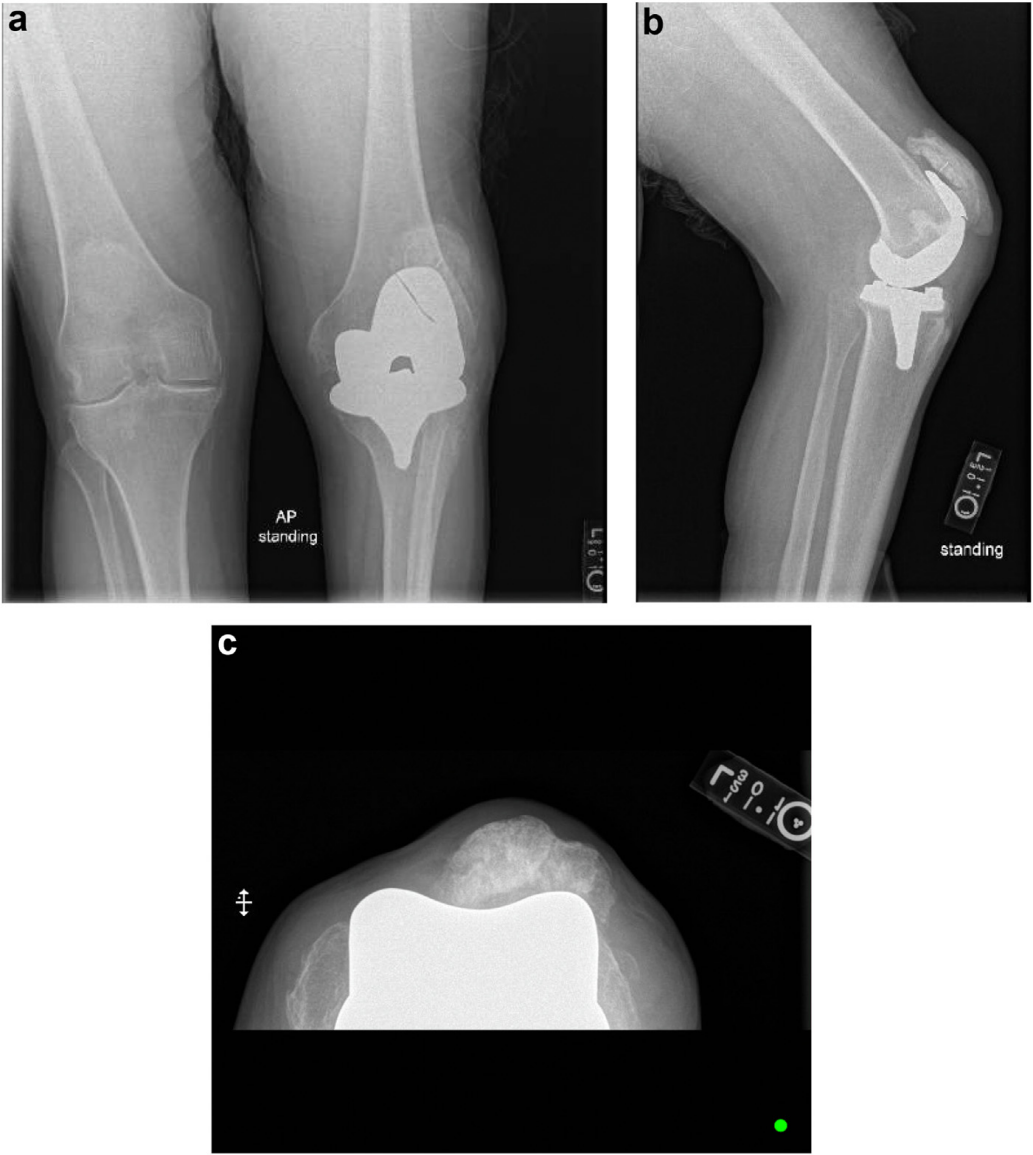
(a) Anteroposterior, (b) lateral, and (c) sunrise radiographs of left knee on presentation 12 years after primary TKA.

In 2010, he underwent an uncomplicated primary TKA due to osteoarthritis of the left knee. At the time of the primary TKA, there was an attempted hamstring release; however, the contracture returned. He had been functioning well until 3 months prior to presentation, when he developed pain in his knee. He denied any recent falls or trauma. At the time of presentation, his pain was reported to be 3/10 on the numerical rating scale, although it increased to 7/10 with activity. Physical exam showed ambulation with an altered gait and 30-degree flexion contracture. He had a high riding patella, but it tracked appropriately. His knee was ligamentously stable and neurovascularly intact with no signs of infection, and inflammatory markers were within normal limits. Radiograph imaging showed a fractured femoral component at the anterior flange (Fig. 1a-c). He was indicated for revision TKA. Aside from the subdural hematoma that led to his persistent neuromuscular flexion contracture, our patient had no other relevant past medical history or comorbidities to report. His body mass index at the time of presentation was 31.5 kg/m^2^.

**Figure 2:**
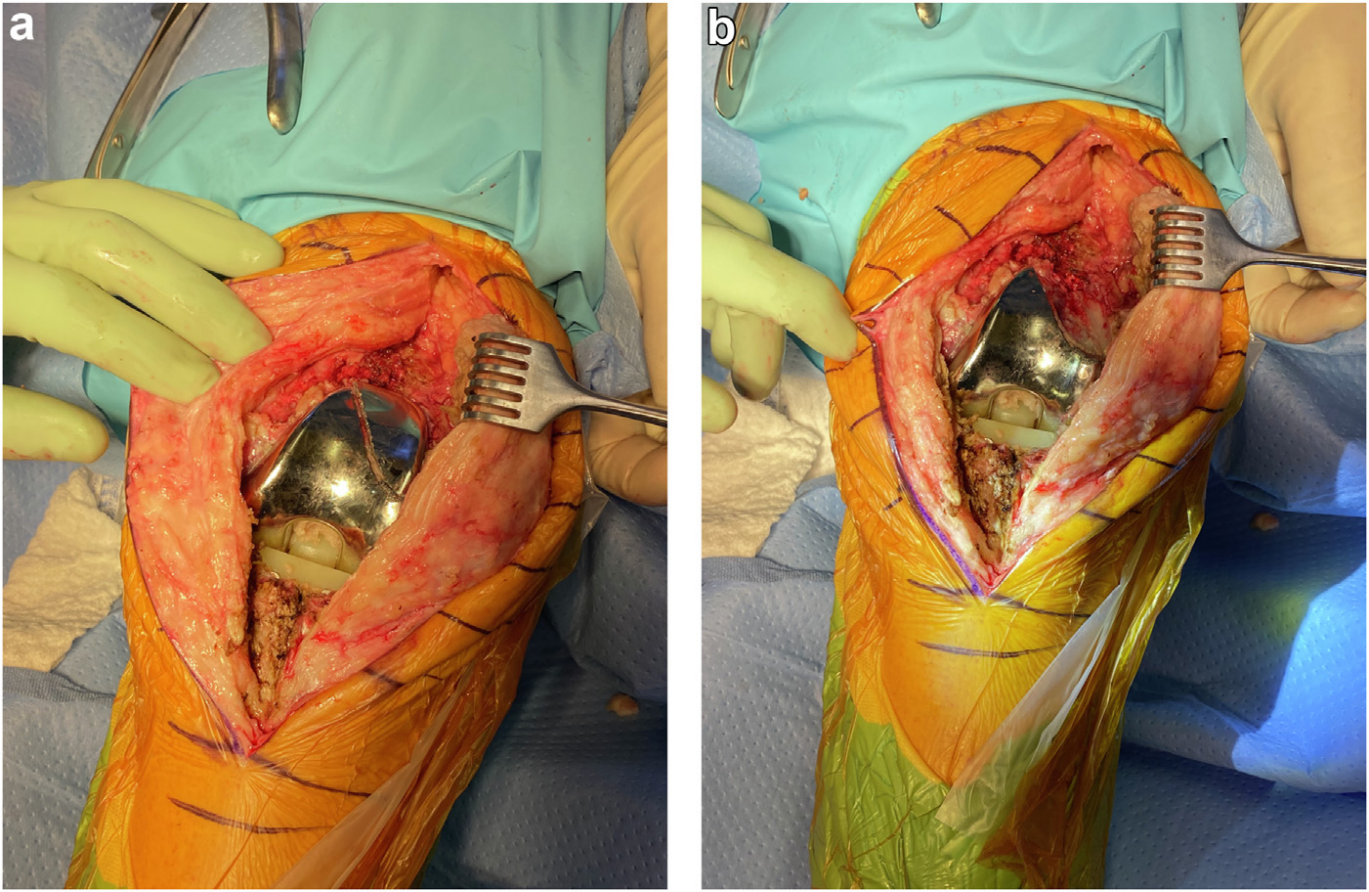
(a) Intraoperative views of fractured anterior flange. (b) Intraoperative view after removal of the fractured anterior flange.

**Figure 3:**
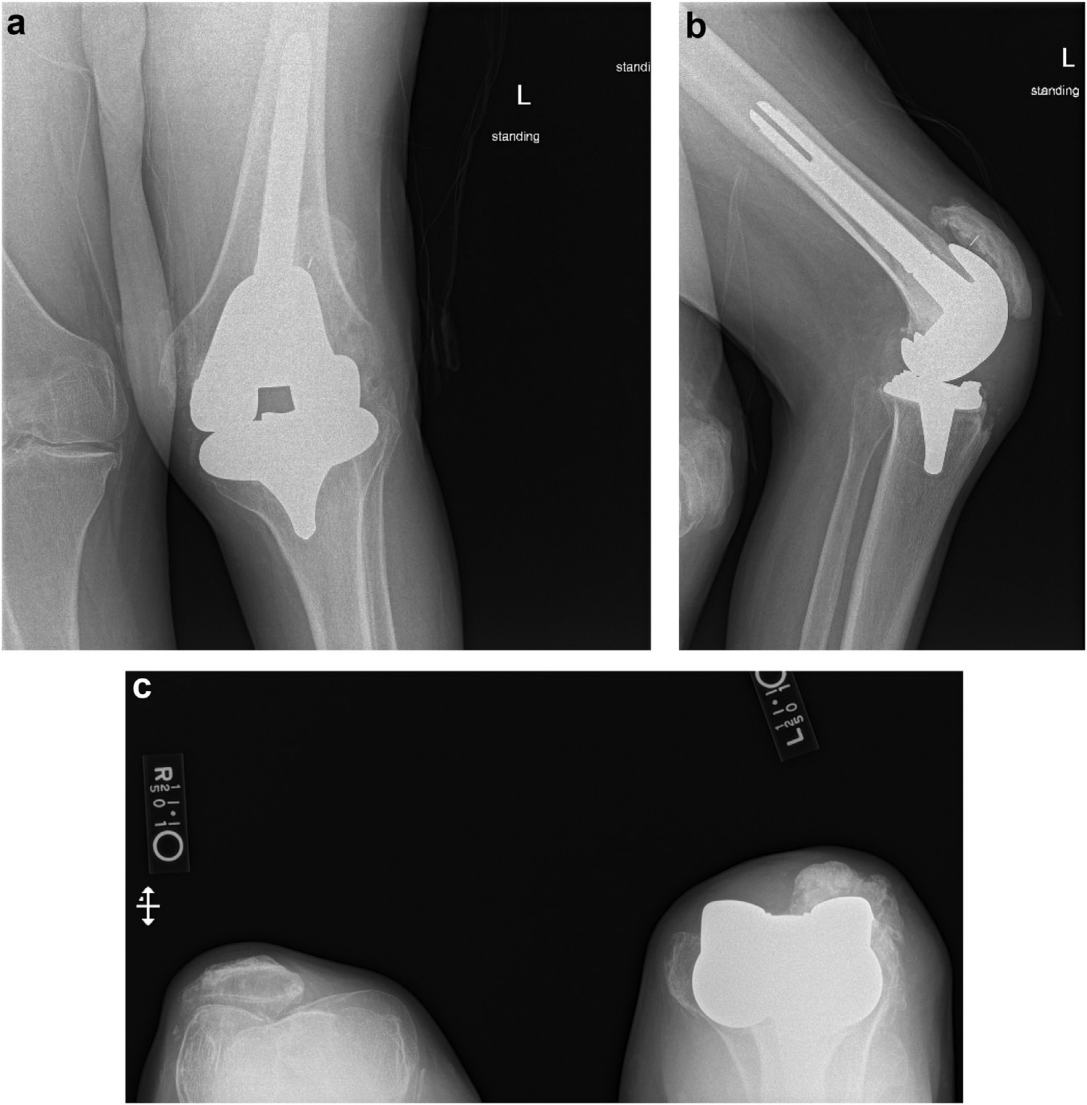
(a) Anteroposterior, (b) lateral, and (c) sunrise postoperative radiographs of left knee 6 months after revision surgery.

During the revision, we confirmed the anterior flange fracture of the Vanguard knee prosthesis (Fig. 2a and b). Aside from the obvious fracture, the femoral component showed no signs of damage; it was well cemented and stable. Despite being fractured, the anterior flange was well fixed. It was removed with minimal bone loss, and residual cement was also removed. There was also no indication of any underlying cantilevering mechanism or osteolysis. Upon inspection, the tibial component was well preserved, not loose, and maintained locking mechanism integrity without scratches or other signs of damage. We proceeded with the revision of the femoral component only. We also decided not to address the flexion contracture due to previous failed attempts and because he was happy and functioning well with his prior range of motion, as this had been his level of function for his entire life. We performed a revision of the femoral component taking care to fully reinforce the anterior flange by placing it directly on the anterior cortex of the femur. Postoperative recovery was uncomplicated. Postoperative radiographs from the 6-month follow-up visit showed a well-seated component with no concerns (Fig. 3a-c). At 1 year postoperation, the patient had no pain in his left knee, and his flexion contracture was maintained at 30 degrees. There were no other complications to report at the 1-year mark.

## Discussion

The majority of reported femoral component failures have occurred in the Ortholoc II (Wright Medical, Memphis, TN). Four authors have documented a total of 43 cases of fatigue failure of the Ortholoc II TKA femoral component [[2], [3], [4], [5]]. The average time to failure for these components was 27.3 months. These failures have been attributed to a design flaw where the portion of the implant that sits on the posterior chamfer cut is too thin, leaving it susceptible to fatigue failure [1].

Outside of the Ortholoc II failures, there have been a limited number of other cases reported in the English literature. These cases occurred in a variety of TKA systems with no identifiable pattern and were split between cemented and uncemented components [1,[6], [7], [8], [9], [10], [11], [12], [13], [14]]. The uncemented failures were attributed to uneven bony surfaces due to poor saw cuts or osteolysis, which led to uneven load distribution and fatigue failure. The average time to failure for uncemented femoral components was 84 months. The cemented failures were attributed to aseptic loosening or poor cementing technique, which left the components vulnerable to fatigue failure. The average time to failure for the cemented components was 80.2 months [1].

The previously reported component fractures have been attributed to fatigue failure due to poor underlying support. To our knowledge, all other failures have occurred at or near the medial or lateral femoral condyles. This is understandable given that these are the areas of high stress, especially when coupled with poor underlying support. The fractured implant in our patient was the Vanguard knee prosthesis; to our knowledge, there has been only one other reported fracture of these implants [15]. This component failure occurred 10 years after implantation. During explantation, the authors identified a fracture on the superolateral aspect of the femoral component’s anterior flange. Under closer examination, there were no signs of osteolysis, and there was a lack of cement underneath the fractured fragment. The authors attributed the fracture to fatigue failure secondary to poor cementing technique.

Here, we report a femoral component failure with a fracture of the anterior flange, likely due to increased stress secondary to a persistent flexion contracture. The femoral component was likely subjected to increased pressure from the high riding patella due to the flexion contracture. The lateral radiograph shows the patella resting directly over the fracture site. Over the course of 12 years, this led to fatigue failure. In other similar cases reported in the literature, poor cementing, uneven cuts, or osteolysis contributed to a femoral component fracture. These scenarios could result in a cantilevering situation, which would cause the component to be more susceptible to failure. In our case, there was no sign of improper cementing or osteolysis. On explantation, the anterior flange fracture fragment was backed with a cement mantle. The anterior flange was minimally displaced, as seen on the lateral radiograph as well as intraoperatively. There was no radiographic or intraoperative evidence of any cantilevering mechanisms. The lack of these findings supports our hypothesis of fatigue failure due to the increased pressure from the patella. Furthermore, the other reported failures due to operative factors failed on average much sooner than the component reported here. The average time to fail is about 7 years, and our component failed after 12 years. This supports our hypothesis that our component was properly seated with an adequate cement mantle and that the failure was in fact due to fatigue. In patients with abnormal load distributions, such as obesity or contractures, it is essential to place the implants on a supportive, even base of bone for cementless implants or cement for cemented implants in order to decrease the risk of fatigue failure.

Despite our previously stated hypothesis, the exact cause of the failure cannot be determined without a metallurgical analysis of the component to rule out material discontinuity or surface defect. Casting defects or metallurgical flaws would certainly contribute to failure. However, given the location of the fracture directly under the high riding patella, the lack of suspicion for cantilevering forces, and the fact that the failure occurred 12 years after implantation, we believe that the failure was a result of fatigue. If a material defect were present, its manifestation was likely hastened by the increased fatigue from the patient’s altered gait, spasticity, flexion contracture, and patella position.

## Summary

Although femoral component fractures are rare, they should be considered as a potential complication, especially in patients with special load considerations. For these patients, it is essential that the prosthesis be properly supported with clean cuts and an adequate cement mantle.

## Conflicts of interest

The authors declare there are no conflicts of interest.

For full disclosure statements refer to https://doi.org/10.1016/j.artd.2023.101174.

## Informed patient consent

The author(s) confirm that written informed consent has been obtained from the involved patient(s) or if appropriate from the parent, guardian, power of attorney of the involved patient(s); and, they have given approval for this information to be published in this case report (series).

## Appendix A Supplementary data

Conflict of Interest Statement for Schneider

Conflict of Interest Statement for Tate

Conflict of Interest Statement for Brown
