# Supplementary material for: Femoral Component Fracture in a Total Knee Arthroplasty Patient With a Persistent Flexion Contracture
Source: Arthroplast Today. 2023 Jul 22;22:101174. doi: 10.1016/j.artd.2023.101174 (PMC10374856; doi:10.1016/j.artd.2023.101174)
Supplement: Conflict of Interest Statement for Brown [file mmc3.docx]

# INDIVIDUAL CONFLICT OF INTEREST STATEMENT

***American Association of Hip and Knee Surgeons***

(Adopted from the American Academy of Orthopaedic Surgeons disclosure statement)

The following form **must be filled out completely and submitted by each author (example, 6 authors, 6 forms).**

**All items require a response. If there is no relevant disclosure for a given item, enter "*None*.”**

Femoral Component Fracture in a Total Knee Arthroplasty Patient with a Persistent Flexion Contracture

**Manuscript Title**

1. Royalties from a company or supplier (The following conflicts were disclosed)

none

2. Speakers bureau/paid presentations for a company or supplier (The following conflicts were disclosed)

none

3A. Paid employee for a company or supplier (The following conflicts were disclosed)

none

3B. Paid consultant for a company or supplier (The following conflicts were disclosed)

Depuy, Corin

3C. Unpaid consultants for a company or supplier (The following conflicts were disclosed)

none

4. Stock or stock options in a company or supplier (The following conflicts were disclosed)

none

5. Research support from a company or supplier as a Principal Investigator (The following conflicts were disclosed)

none

6. Other financial or material support from a company or supplier (The following conflicts were disclosed)

none

7. Royalties, financial or material support from publishers (The following conflicts were disclosed)

none

8. Medical/Orthopaedic publications editorial/governing board (The following conflicts were disclosed)

none

9. Board member/committee appointments for a society (The following conflicts were disclosed)

AAOS OKU evaluation committee, AAOS AJRR committee

**Each author must sign AND print or type his/her name, date and submit a separate form**

In addition, one BLINDED Conflict of Interest form (no author names used) should be submitted per manuscript with all author disclosures.


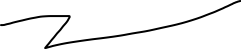


Nicholas Brown 4.4.2023

Author Name (Print or Type) Author Signature Date
